# ANTIMICROBIAL RESISTANCE AND MORTALITY

**DOI:** 10.21010/Ajid.v16i2.2

**Published:** 2022-05-06

**Authors:** Hori Hariyanto, Corry Quando Yahya, Cucunawangsih Cucunawangsih, Cecilia Lenny Pravita Pertiwi

**Affiliations:** a Universitas Pelita Harapan-Faculty of Medicine, Department of Anesthesiology and Intensive Care, Jl. M. H. Thamrin Boulevard 1100, Lippo Village Tangerang,Tangerang, Banten 15811, Indonesia; b Universitas Indonesia-Faculty of MedicineJalan Diponegoro No 77, Jakarta Pusat 10430 Indonesia; c Universitas Pelita Harapan-Faculty of MedicineDepartment of Microbiology, Jl. M. H. Thamrin Boulevard 1100, Lippo Village Tangerang,Tangerang, Banten 15811, Indonesia; d Siloam Hospitals Lippo VillageJl. Siloam No.6Karawaci, Tangerang, Banten 15811 Indonesia

**Keywords:** multi-drug resistance, antibiotic resistancy, mortality, virulency, infection

## Abstract

**Background:**

Antibiotic resistance has been a long-debated topic since decades ago. The development of stronger, newer antibiotics, implementation of antibiotic stewardship and revised guidelines remain the main focus of our society to prevent resistancy. But is it really resistancy that cause higher mortality to patients with multidrug resistance (MDR) infections?

**Methods:**

We conducted a cohort retrospective study from 2016 to 2019 in our Intensive care unit (ICU). Antimicrobial susceptibility test (AST) results were analyzed for their association with patient mortality outcomes.

**Results:**

Over the four-year period, 381 positive bacterial cultures were analyzed and 51% of them grew MDR pathogens upon their first culture. The overall mortality rate was 19% (38/195), and there was no significant association between MDR and mortality; *p* 0.387. A strong association was however found between patients with medical cases with an OR 1.76; CI 1.76-2.55; *p* 0.003 and those with APACHE scores ≥20 upon admittance to the ICU, OR 1.32; CI 1.68-8.29; *p* 0.001.

**Conclusion:**

Resistancy is not the true cause of mortality. Infection by resistant microbes does not necessarily mean the worst outcome since virulency is the actual cause of pathogenicity, and thus mortality.

## Introduction

Antibiotics have offered mankind a dramatically-new approach to infection control and profound reduction in mortality and morbidity against various infectious diseases. Over time, some of these bacteria have evolved to create new species of multi-drug resistant (MDR) pathogens and fought back. While antimicrobial agents play a key role in controlling and curing infectious disease, are they a major determinant to the high mortality rate encountered in patients with MDR infections? For many years, clinicians have shared the paradigm that infections with antimicrobial resistance were doomed with poor outcome and mortality. However, assessment of the contribution of MDR infections to adverse clinical outcome is difficult, given the confounding factors such as illness severity, co-morbidities, infection site and treatment strategies which may affect mortality, too.

Since 2014, resistance to key antibiotics have been reported amongst 6 World Health Organization (WHO) work areas in the regions of Africa, the Americas, eastern Mediterranean, Southeast Asia, Europe, and the Western Pacific with 50% of *Escherichia coli*, *Methicillin Resistant Staphylococcus aureus* (MRSA), and *Klebsiella pneumonia* that are resistant to third-generation cephalosporins and fluoroquinolones. (World Health Organization 2014) A large-scale study (EUROBACT) on nosocomial bloodstream infections conducted amongst 24 Intensive care units (ICU) in 2018 worldwide stated an average MDR rate of 47.8%; 20.5% of extensively drug-resistant (XDR) and 0.5% of pan-drug-resistant (PDR) patterns. Striking variability were also found among different countries ranging from as low as 8% (Australia); 59.3% in Asian countries (Kang and Song 2013) to more than 70% resistance (Greece, Turkey, Serbia, Croatia, Morocco). (Tabah *et al*. 2012) Mortality from cultured MDR strains ranges from 5% to 17% in European countries (de Kraker *et al*. 2011); while Asian countries reported a staggering 46% to 67% mortality rate. (Kang and Song 2013)

Interestingly, amongst those with positive association, some were found as an indirect consequence to inappropriate antimicrobial therapy or due to increased length of stay (Dautzenberg *et al*. 2015), while another found that antimicrobial resistance had a low effect on mortality. (Lambert *et al*. 2011) At present, the association between MDR infections and mortality remains controversial. Several studies have reported a direct association whereas others have shown no association between MDR infections and mortality. (Paramythiotou and Routsi 2016) Taking this into account, our study aims to look at the impact of MDR infections upon mortality rate, with particular emphasis to the ICU population.

## Materials and methods

### Study design and data collection

A cohort retrospective study was conducted between January 2016 and January 2019, among ICU patients in Siloam Hospital Lippo Village, Indonesia. Data were extracted and collected from medical record. We collected information on baseline characteristics, laboratory tests, treatment, procedures, and outcomes. Only the first positive culture (MDR or non-MDR Gram positive and/or Gram negative) in the specimen of each patient was included in our analysis. Readmission was excluded and only the first hospitalization of each patient was included. Patients with automatic discharges were excluded on the analysis of the outcome. All antimicrobial susceptibility testing (AST) were obtained in the ICU and mortality data were restricted to 30-day all-cause mortality in the ICU.

### Definitions

Diagnostic criteria for different types of infection were adopted from the Center for Disease Control and Prevention (CDC). A bloodstream infection (BSI) is defined as one or more positive blood cultures associated with systemic signs of infection such as fevers, chills, and/or hypotension.

A sputum culture is a microbiology test performed to isolate and identify microorganisms causing an infection of the lower respiratory tract in association with new or progressive pulmonary infiltrates on chest X-rays; new onset or worsening cough or dyspnea or tachypnea; or worsening gas exchange.

Urinary tract infection (UTI) is defined as a positive urine culture of ≥10^5^ colony forming units/ml and with no more than two species of microorganisms, and at least one of following signs or symptoms: fever (> 38°C); dysuria; suprapubic tenderness; costovertebral angle pain or tenderness with no other recognized cause.

Pus is defined as purulent drainage obtained from sites such as the skin, subcutaneous tissue or deep soft tissue of the incision with or without laboratory confirmation; organisms isolated from an aseptically obtained culture of fluid or tissue; or one of the signs or symptoms of infection: pain or tenderness, localised swelling, redness, or heat.

Multi-drug resistance is defined based on in-vitro antimicrobial susceptibility test results, when isolates are ‘resistant to three or more antimicrobial classes’ according to Macgiorakos et al. (Magiorakos *et al*. 2012).

Continuous variables were presented as mean ± standard deviation (SD) while categorical variables were shown as counts or counts/total (percentages). Statistical comparisons were performed using the Student’s t-test or Mann–Whitney U-test, chi-square

### Microbiology

All isolates were grown and identified by VITEK 2 system (bioMérieux, Marcy l’Etoile, France) to conduct organism identification and antimicrobial susceptibility test (AST). *Escherichia coli* ATCC 25922 and *Staphylococcus aureus* ATCC 25923 was used as a quality control reference strain. The results were interpreted in accordance with the recommendations of the Clinical and Laboratory Standards Institute (CLSI2018).

### Statistical analysis

Tests or fisher’s exact test, were done as appropriate. Risk factors for mortality were explored using multivariate logistic regression and the results were listed as odds ratio (95% confidence interval). Statistics were analysed using SPSS 16.0 software and two-sided significance level of 0.05 was selected.

### Ethical Considerations

All patients completed an informed consent form that was approved by the Ethical Committee at Siloam Hospitals, Lippo Village (Study protocol: 19-03-0317).

## Results

**Figure 1:**
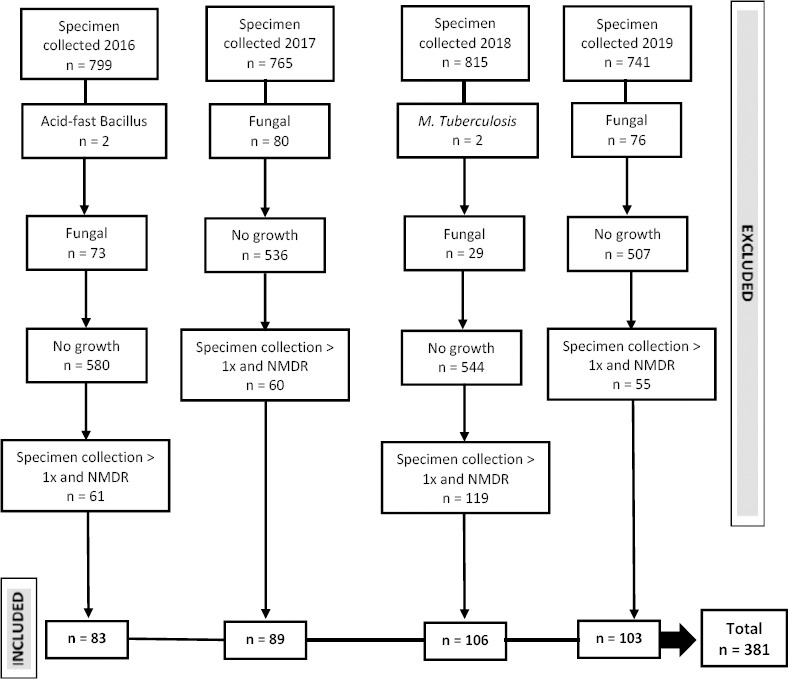
Flow diagram of the study population

A total of 3,120 patients were admitted to the ICU during the 4-year study period (2016-2019). After elimination through exclusion criteria, we had a total of 381 samples. Figure 1.

The mean age was 57±19 years with males comprising majority of the ICU population (65% vs. 35%) and 27% of them had some form of comorbidity. Our ICU bed admittance was divided into surgical vs medical cases (23% vs 77%). Apache scores greater than 20 comprised majority of the ICU population (73%).

Specimen source were collected from various sites and bacteria were most frequently isolated from sputum (265/381, 70%) followed by blood stream (BSI) (74/381, 19%), pus (29/381, 7%), cerebrospinal fluid (8/381, 2%) and urine (5/381, 1%) samples. Majority of the isolate cultured were Gram negative (84.5%) vs Gram positive (15.5%) with as many as half of these isolates grew MDR pathogens (51%). The average length of stay (LOS) was 9±8 days for all patients; surviving patients had a mean LOS of 9±6 days compared to non-survivors of 7±7 days. In this study, we found half of the population acquired MDR infection (195/381, 51%); nevertheless, majority of them survived (157/195, 81%) as compared to those who died (38/195, 19%). Table 1.

**Table 1:** Baseline and clinical characteristics of patients

Variable	All		Survivors		Non-survivors	
	n	%	n	%	n	%
**Gender**						
Male	246	64.6	196	65.6	50	61.0
Female	135	35.4	103	34.4	32	39.0
**Age (years)**	57.7±19.3		56.8±19.5		61.1±7.9	
Mean±SD	0±96		0±95		2±96	
**Comorbid**						
Present	104	27.3	75	25.1	29	35.4
Not present	277	72.7	224	74.9	53	64.6
**Case**						
Surgical	88	23.1	81	27.1	7	8.5
Medical	293	76.9	218	72.9	75	91.53
**APACHE Score**						
Mean±SD	24.7±7.6		23.3±6.8		30.1±8.2	
≥20	266	73.5	196	69	70	90
<20	96	26.5	88	31	8	10
**LOS (days)**						
Mean±SD	8.6±6.7		9.2±6.5		6.9±7.3	
Min±Max	0±46		0±46		0±33	
**Specimen Source**						
Sputum	243	63.8	188	62.9	55	67.1
Blood	74	19.4	54	18.1	20	24.4
Pus	29	7.6	26	8.7	3	3.7
Bronchial Fluid	22	5.8	20	6.7	2	2.4
Cerebrospinal Fluid	8	2.1	6	2.0	2	2.4
Urine	5	1.3	5	1.7	0	0
**Isolates**						
Gram Negative	322	84.5	249	83.3	73	89.0
Gram Positive	59	15.5	50	16.7	9	11.0
**Multi-drug resistant (MDR)**						
Yes	195	51.2	157	47.5	38	46.3
No	186	48.8	142	52.5	44	53.7

APACHE = Acute Physiology and Chronic Health Evaluation; LOS = length of stay; MDR = Multi-drug resistance.

Bivariate analysis on the relationship between MDR and mortality was 0.78 (95%, CI: 0.479-1,257). This meant that MDR infection actually confer a protective effect from mortality. However, chi-square test did not reveal a significant relationship between MDR and survival rates in our ICU, p = 0.387. Gram negative isolate comprised the majority isolate in our center and was found to have no significant relationship with mortality; OR of 1.629 (95%, CI: 0.765-3,469), p = 0.203. Hence, neither the isolate type nor the specimen source had a significant association with mortality. In addition, the presence of comorbid did not convey any relationship to mortality in the ICU, p = 0.066. Table 2

**Table 2:** Bivariate logistic regression for variables associated with hospital mortality

Variable		Survivors		Non survivors		*p-value*	OR (95%CI)

		n	%	n	%		
**Age (years)**							
	≥55	185	75.2	61	24.8	0.037	1.78 (1.03-3.09)
	<55	144	84.4	21	15.6		
**Comorbid**							
	Yes	75	72.1	29	27.9	0.066	1.63 (0.96-2.75)
	No	224	80.9	53	19.1		
**Case**							
	Surgical	81	27.1	7	8.5	0.001	3.98(1.76-8.99)
	Medical	218	72.9	75	91.5		
**APACHE score**							
	≥20	196	69	70	89.7	0.001	3.92 (1.81-8.51)
	<20	88	30.1	8	1.3		
**Specimen source**						0.214	
	Sputum	188	62.9	55	67.1		
	Blood	54	18.1	20	24.4		
	Pus	26	8.7	3	3.7		
	Bronchial Fluid	20	6.7	2	2.4		
	CSF	6	2	2	2.4		
	Urine	5	1.7	0	0		
**Isolate**							
	Gram negative	249	83.3	73	89	0.203	1.629 (0.76-3.46)
	Gram positive	50	16.7	9	11		
**MDR**							
	Yes	157	52.5	38	46.3	0.387	0.781 (0.479-1.25)
	No	142	47.5	44	53.7		

APACHE = Acute Physiology And Chronic Health Evaluation; MDR = Multi-drug resistance.

On the contrary, this study found a significant association between older age classified as ≥55 years old and mortality. Moreover, case classification and mortality had an OR 3.98 (95%, CI: 1.76-8.99), p = 0.001 which meant that the odds of mortality to patients from the medical ICU were 3.9 times higher compared to those from the surgical ICU. Another significant relationship was found between Apache score and mortality, p = 0.001. In this study, an APACHE cut-off score of ≥ 20 was used with 85.37% sensitivity, and 35.45% specificity.

Further analysis using multivariate study revealed no significant association between mortality to age or comorbid status. After controlling for age, case classification, comorbid status, Apache score and MDR infection status, we found an association between APACHE II score and mortality with p value of 0.001, OR 3.74 and 95% CI (1.68-8.29).

This meant that patients admitted to the ICU with an APACHE score ≥ 20 had a 3.7 times greater chance of dying as compared to those with APACHE score less than 20.

Another variable significantly associated with mortality are patients admitted as medical case classification, OR 1.76 (95% CI 1.21-2.55). Hence, admission from medical cases to our center had a 1.8 times higher risk of mortality compared to those admitted from surgical cases. Surprisingly, multi-drug resistance and mortality seemed to confer a protective effect given its beta coefficient value of -0.373, with an OR 0.68 and *p* value of 0.164. Table 3

**Table 3:** Multivariate analysis for variables associated with hospital mortality

Variable	Coefficient	*p value*	OR	95% CI
**Age (years)**	0.2006	0.525	1.22	0.65; 2.27
**Case (Medical/Surgical)**	0.563	0.003	1.76	1.21; 2.55
**Comorbid**	0.3005	0.292	1.35	0.77; 2.36
**APACHE score (cut off 20)**	1.319	0.001	3.74	1.68; 8.29
**MDR**	-0,373	0.164	0.68	0.41; 1.16

## Discussion

Microorganisms have always lived amongst us. Of all the cells that make up the normal, healthy human body, more than 90 percent of them are microbes that live on the skin or in the gut and represents the normal microbial population with some potential pathogens. (Turnbaugh *et al*. 2007) Evidently, nosocomial infections are caused by these natural inhabitants that have evolved to adapt to its changing environment. Resistancy to antibiotics holds the same phenomenon. This defensive mechanism began to develop after the introduction of antibiotics, about 50 years ago.

(Beceiro, Tomás, and Bou 2013) Even then, persistence of these resistant pathogens continue to develop through hypermutation, compensatory mutations and cross selection despite absent or minimal concentration of antibiotics. (Andersson and Hughes 2011) (Livermore 2003). Knowing this, are antibiotics to blame for the appearance of resistant microorganisms? More importantly, does resistancy birth ‘superbugs’ that causes higher mortality?

A deeper understanding on bacterial resistancy and bacterial virulency is discussed, herein. Resistancy is the ability to withstand antibiotic bacteriostatic and/or bactericidal properties, while virulency are the factors that make a microorganism harmful to its host. Resistance to antibiotics maybe one of the several virulence properties a microbe possesses; but resistancy does not always confer to pathogenicity which is the ability to inflict an infectious process.

Likewise, an increase in resistancy may produce an increase, decrease or no effect on virulency. (Cepas and Soto 2020) An example is seen in the overexpression of MexAB-OprD gene, an efflux pump in *Pseudomonas aeruginosa* that are able to export quinolones, tetracyclines, chloramphenicol and beta lactams, thus conferring resistance but at the same time decreases the production of proteases and its virulence factor. *(Linares et al*. 2005).

Virulency is determined by two factors. The first is toxicity which is defined as the degree to which a microbe causes harm through special proteins, pili, adhesins, exotoxins or endotoxins. (Cepas and Soto 2020) One well-known toxin producing bacteria is Enterotoxigenic *Escherichia coli* (ETEC) which releases enterotoxins to increase the concentration of cyclic adenosine monophosphate (cAMP) and cyclic guanosine monophosphate (cGMP) in enterocytes, resulting in the massive efflux of electrolyte-rich fluid into the intestinal lumen manifested as profuse watery diarrhoea. (Fleckenstein *et al*. 2009) On the contrary, Enteropathogenic *Escherichia coli* (EPEC) uses a protein, Tir, that inserts itself into the host cell membrane where it acts as a receptor for a bacterial adhesin, intimin. Binding of intimin to Tir results in cytoskeletal rearrangements characterized by a decrease in the number and height of microvilli, blunting of enterocyte borders, loss of glycocalyx and presence of a mucous pseudo membrane coating the mucosal surface and manifests as severe watery diarrhoea and fever leading to considerable mortality (Levine 1987).

The second factor is invasiveness, which is the ability to penetrate into the host and spread. They are mediated by invasins, capsules and biofilm formation. An example is *Pseudomonas aeruginosa* with an overexpression of type III secretion system (TTSS) leading to an increase in bacterial fitness and virulence, thus increasing colonisation and systemic dissemination, even under low pH. (Roux *et al*. 2015) Another form of invasiveness is the ability of microbes to form biofilm which acts as a deterrent to the immune system and breeding ground for colonies to divide and transfer virulence genes. In this way, microorganisms containing antimicrobial resistance or virulence genes can contact with non-resistant cells and transfer genetic materials vital to the establishment of new resistant virulent cells. (Schroeder, Brooks, and Brooks 2017).

Antibiotic resistance is a well-known occurrence amongst ICU population as they are the last dwelling place for the sickest of sick patients, but one should bear in mind that resistancy does not equal virulency, which is the ability to cause harm, and thus mortality.

Our study found no association between antimicrobial resistancy and mortality. Likewise, a large study involving 1265 ICUs from 76 countries: The Extended Prevalence of Infection in Intensive Care (EPIC II) has shown that antimicrobial resistance in the ICU setting is not always associated with the worst outcome. (Hanberger *et al*. 2014). A literature review involving 24 studies on the ICU population worldwide found non-consistent results of MDR infections and mortality. (Paramythiotou and Routsi 2016) Blot *et al*. found that antibiotic resistance in nosocomial bacteraemia caused by gram-negative bacteria does not adversely affect the outcome for critically ill patients. (Blot *et al*. 2002) In a Finnish cohort study, Kontula *et al*. stated that causative pathogens of nosocomial blood-stream infection and MDR bacteria cannot be interpreted as a risk for severe outcome in patients. (Kontula *et al*. 2018) In fact, studies on the microbial genome suggested bacterial genotyping to be highly predictive factors for adverse infection outcome and mortality compared to other factors such as patient age, gender or comorbidities. (Recker *et al*. 2017) (Horváth *et al*. 2020) Hence, virulency is key to producing a lethal effect, not resistancy.

Aside from resistancy and virulency, the host-pathogen interaction is crucial to the development of an infection and therefore in how the acquisition of resistance affects virulence. (Casadevall and Pirofski 2000) In this study, mortality was significantly associated with medical cases and those with APACHE score greater than 20. It is not surprising as majority of patients from the medical ward have been hospitalized longer, acquire multiple comorbidities and had former history of antibiotic usage compared to those from surgical ward. Once admitted to the ICU, they are subjected to multiple invasive procedures, use of sedatives, analgetics and stress-ulcer prophylaxis: all of which compromise and impair protective reflexes such as cough reflex, skin protective barrier, disruption of normal gastric acidity, decreased gut-motility leading to colonization and translocation of pathogenic bacteria into sterile sites. (Khan, Baig, and Mehboob 2017).

Given the high rates of positive cultures caused by MDR organisms, we sought to examine the most common causative organism and found majority of our isolate to be gram negative with 51% acquiring an MDR status. Similar studies from India had identified 58% of gram negative MDR isolates from its ICU specimens (Subhedar and Jain 2016) while another study from India on ventilator-associated pneumonia found 88% of its isolate to be gram negative with 72% MDR status. (Gupta *et al*. 2017) Such finding matches a systematic review involving 22,876 ICU patients from 7 Southeast Asian countries with a higher incidence of MDR gram negative *Acinetobacter baumannii* (58%) and Carbapenem-resistant antibiotic (65%). (Teerawattanapong *et al*. 2018) Nevertheless, we did not find any significant association between isolate type and mortality.

Our study has the following limitations: Identification of bacterial virulency through polymerase chain reaction (PCR) were not performed in this study. Hence virulency data on MDR organisms were not analysed. Information on previous antibiotic usage prior to ICU admittance were not extracted from each patient. Six percent of our study population comprised of the pediatric population (0-17years old) and were not analysed for their APACHE score; however, data on their culture and antibiotic susceptibility test were included for analysis.

## Conclusion

Antimicrobial resistance is a natural phenomenon much like the evolution of living beings adapting to its ever-changing environment. Bacteria resistant to an antibiotic (naturally or by an acquired mechanism) are normally selected by the pre-existence of low-level resistant microorganisms able to survive under such conditions. As a result, resistant microbes may be routinely cultured from patients, but their clinical condition does not always correlate to being “infected”. Resistancy and virulency are distinct properties of a microbe. Virulency inflicts host cell damage, while resistancy is a protective defence mechanism. To this aim, special attention ought to be targeted to virulency which are the true cause of pathogenicity. Together with the best antibiotic strategy and growing understanding on microbial properties, we hope to win the fight against infections of MDR bacteria, in the near future.

### Conflict of Interest

The authors declare that they have no competing interests associated with this study.

**List of Abbreviations:**
Multidrug resistance =: MDR
*Methicillin Resistant Staphylococcus aureus* =: MRSA
Extensively drug-resistant =: XDR
Pan-drug-resistant =: PDR
Antimicrobial susceptibility testing =: AST
Bloodstream infection =: BSI
Length of stay =: LOS
Enteropathogenic *Escherichia coli* =: EPEC
